# Same Modulation but Different Starting Points: Performance Modulates Age Differences in Inferior Frontal Cortex Activity during Word-Retrieval

**DOI:** 10.1371/journal.pone.0033631

**Published:** 2012-03-15

**Authors:** Marcus Meinzer, Tobias Flaisch, Lauren Seeds, Stacy Harnish, Daria Antonenko, Veronica Witte, Robert Lindenberg, Bruce Crosson

**Affiliations:** 1 Department of Clinical and Health Psychology, University of Florida, Gainesville, Florida, United States of America; 2 Department of Neurology, Center for Stroke Research Berlin and Cluster of Excellence NeuroCure, Charite Universitätsmedizin, Berlin, Germany; 3 Department of Psychology, University of Konstanz, Konstanz, Germany; 4 Brain Rehabilitation Research Center, Malcom Randall VA Medical Center, Gainesville, Florida, United States of America; 5 Department of Speech, Language, and Hearing Sciences, University of Florida, Gainesville, Florida, United States of America; University Of Cambridge, United Kingdom

## Abstract

The neural basis of word-retrieval deficits in normal aging has rarely been assessed and the few previous functional imaging studies found enhanced activity in right prefrontal areas in healthy older compared to younger adults. However, more pronounced right prefrontal recruitment has primarily been observed during challenging task conditions. Moreover, increased task difficulty may result in enhanced activity in the ventral inferior frontal gyrus (vIFG) bilaterally in younger participants as well. Thus, the question arises whether increased activity in older participants represents an age-related phenomenon or reflects task difficulty effects. In the present study, we manipulated task difficulty during overt semantic and phonemic word-generation and used functional magnetic resonance imaging to assess activity patterns in the vIFG in healthy younger and older adults (N = 16/group; mean age: 24 vs. 69 years). Both groups produced fewer correct responses during the more difficult task conditions. Overall, older participants produced fewer correct responses and showed more pronounced task-related activity in the right vIFG. However, increased activity during the more difficult conditions was found in both groups. Absolute degree of activity was correlated with performance across groups, tasks and difficulty levels. Activity modulation (difficult vs. easy conditions) was correlated with the respective drop in performance across groups and tasks. In conclusion, vIFG activity levels and modulation of activity were mediated by performance accuracy in a similar way in both groups. Group differences in the right vIFG activity were explained by performance accuracy which needs to be considered in future functional imaging studies of healthy and pathological aging.

## Introduction

Word-retrieval difficulties are frequent in healthy aging and age-related pathological processes (e.g., dementia and its precursors [1]; post-stroke aphasia [2]). However, the neural basis of these impairments is largely elusive and a thorough understanding of activity changes associated with healthy aging is a prerequisite for interpreting functional imaging findings in age-related pathological conditions.

Across cognitive domains, normal aging is frequently associated with less lateralized processing in prefrontal areas [3]. More generally, the hemisphere that is not dominant for a given task might be more active in older compared to younger adults. The functional relevance of this increased activity and the underlying causes have not been conclusively established in the literature so far. However, it is conceivable that structural deterioration of specialized neural populations in the task-dominant hemisphere or of white matter structures connecting those areas with homologous areas may result in reduced inter-hemispheric interplay yielding a disinhibition of contralateral regions [4], [5]. Enhanced task-related activity in older adults has also been interpreted as an effective compensatory mechanism for structural degeneration when associated with superior performance [3]. Moreover, it may reflect greater demands placed on top-down control processes [4] due to deterioration of specialized neural populations (in older adults) or due to increased task demands (in younger and older adults).

More pronounced functional brain activity in the hemisphere that is not dominant for the task in older compared to younger healthy adults has also been demonstrated during language processing. For example, while younger adults typically showed a strongly left lateralized pattern of activity in prefrontal areas during word-retrieval paradigms, several previous studies found additional activity in right prefrontal areas during the same tasks in older adults [6]–[8]. However, increased task difficulty during word retrieval may result in more pronounced activity in younger participants as well, in particular, in left and right ventral inferior frontal gyrus (vIFG) [9], [10]. This finding raises the question of whether more pronounced vIFG activity in previous word-retrieval studies could be the result of increased task demands in older compared to younger participants, as reflected by decreases in accuracy [6] or increases in reaction time [8]. Indeed, in two previous studies we compared semantic and phonemic word-generation tasks between groups of healthy younger and older German [6] and English native speakers [11]. In both studies, more pronounced right-frontal activity was only found when the older participants produced fewer correct responses as compared to the group of younger subjects (i.e., in the semantic task). No differences were found when performance accuracy was comparable between groups (i.e., in the phonemic task).

Thus, activity differences between age-groups may have been confounded by differences in task demands and performance accuracy. This possibility, however, has not been thoroughly addressed in previous functional imaging studies comparing younger and older adults during word-retrieval tasks [6]–[8], even though studies in other cognitive domains have shown that difficulty level modulates age-related differences in brain activity [9]. Moreover, none of the previous studies on word-retrieval that included younger and older adults have specifically addressed whether there are brain areas that are modulated by task demands in a similar way in both age-groups.

Thus, in the present study we explicitly manipulated task difficulty during overt semantic (category based) and phonemic (letter based) word-generation and used functional magnetic resonance imaging (fMRI) to study blood-oxygenation level dependent (BOLD) activity modulations in the vIFG in healthy younger and older adults. While there are differences between those two tasks with regard to the underlying cognitive processes and associated brain activity patterns [12]–[14], both tasks require a strategic search and controlled retrieval of information and have been shown to elicit robust and overlapping left-lateralized activity in ventral prefrontal cortices [12], [15]. Task difficulty was manipulated in two ways: First, within each word-generation task we chose categories or letters to elicit exemplars with two levels of difficulty, which allowed us to compare modulation of activity within the same task. Second, subjects typically produce more correct exemplars during semantic than during phonemic generation [6], [16], providing a second level of task difficulty. We hypothesized that difficulty modulates activity in bilateral ventral prefrontal cortices in both age-groups, even though activity differences between groups may be present at the same level of task difficulty. We also predicted that task difficulty effects (i.e., absolute performance accuracy and differences between easy and difficult task conditions) would predict activity and activity modulation bilaterally in the vIFG.

## Materials and Methods

### Ethics statement

Written informed consent was obtained from all participants prior to study inclusion. The study was approved by the Institutional Review Board of the University of Florida and conducted in accordance with the Helsinki Declaration.

### Participants

Sixteen healthy older and sixteen healthy younger adults were recruited from the University of Florida and Gainesville, Florida communities (older: mean±SD 68.9±5.5 years, range 61–80; younger: 24.0±4.4, range 19–32). Groups were matched for sex (eight females and males in each group) and education (F(1,30) = .09, p = .75; see **Table 1**). All participants were native English speakers and strongly right handed (Edinburgh Inventory; [17]). Data of twenty-eight participants had previously been reported in a manuscript that addressed neural signatures of word-generation but did not specifically assess the impact of task difficulty within and between age-groups [11]. Two additional participants (#15/16) in each age-group were scanned subsequently and included to increase statistical power for the present analyses. None of the participants had previous or current neurological or psychiatric conditions, cardiovascular disease, uncontrolled hypertension or substance abuse as determined by a clinical interview and a standard health questionnaire. No indicators of cognitive impairment were found during cognitive screening (Mini Mental State Examination; [18]: all ≥27/30 points, both old/young: 29.2±0.9) and all participants scored within the normal range of the Beck Depression Inventory [19]. Additional neuropsychological testing assured normal cognitive functioning in the older group. The battery was comprised of the California Verbal Learning Test (CVLT-2; [20]) and the Digit Span subtest of the Wechsler Adult Intelligence Scale (WAIS-R; [21]) as objective tests of memory function. Naming, executive functions for language, and semantic processing were assessed with the Boston Naming Test (BNT; [22]), the Delis–Kaplan Executive Functions System (D-KEFS Verbal Fluency Tests, [23]), the Test of Language Competence (TLC-E, Ambiguous Sentences subtest; [24]), and the Pyramids and Palm Trees Test [25].

**Table 1:** Demographic and psychometric characteristics of the participants.

	YOUNGER GROUP	OLDER GROUP
	(N = 16, 8 females)	(N = 16, 8 females)
**Age** (years)	24.0±4.4	68.9±5.5
**Education** (years)	14.9±0.9	15.6±1.28
**MMSE** (max. 30)	29.3±0.9	29.1±0.9
**Neuropsychological testing**		
D-KEFS		
*semantic fluency (total animals/boys)*	45.4±7.6	41.3±6.3
*phonemic fluency (total F/A/S)*	49.5±7.9	44.1±10.4
Ambigouos sentences (max. 39)	36.2±2.1	34.4±6.6
Pyramids and Palms (max. 52)	50.6±1.2	50.8±1.0
Boston Naming Test (max. 31)	30.1±1.6	30.4±1.1
Digit span		
*Forward (max. 16 points)*	11.6±2.2	11.9±1.6
*Backward (max. 14 points)*	9.9±1.9	8.8±2.0
California Verbal Learning Test (max. 16)		
*correct recall (after learning trial 5)*	14.0±1.9	11.6±2.4*
*short delay free recall*	13.0±2.9	10.1±1.9*
*short delay cued recall*	13.5±2.7	11.8±2.4
*long delay free recall*	12.9±3.4	10.8±3.5
*long delay cued recall*	13.9±2.6	11.6±2.9*
*long delay recognition hits*	15.5±1.0	15.1±0.9

Mean values of raw scores with standard deviations.

* indicate significant differences between age groups at p<.05.

Consistent with previous reports, the younger subjects performed better on three indices of the CVLT and the verbal fluency tests (D-KEFS); however, the latter were not statistically significant. When considering age-corrected norms, the older group performed within normal ranges on all CVLT indices. No significant differences were found between the age-groups on the Digit Span Test. With respect to confrontation naming (BNT) and semantic processing (Ambiguous Sentences, Pyramids and Palm Trees) the groups performed equally well (see **Table 1** for details).

### Experimental task and stimulus characteristics

Details of the design and acquisition parameters have been reported elsewhere [11]. In short, we implemented two overt paced word-generation tasks in the scanner (category based “semantic” and letter based “phonemic” word-generation). Participants were presented various semantic categories or initial letters, and their task was to generate different exemplars for each category or words beginning with a particular letter. Eight different categories and letters were used. The stimuli were presented in blocks of ten consecutive trials of the same category or letter. Stimuli were preselected based on published reports on the effects of category sizes and numbers of possible words beginning with a particular letter [26]–[30]. We chose four easy categories (i.e., many possible exemplars: body parts, clothing, colors, beverages) and letters (many possible words beginning with the respective letter: M, S, T, P) and four difficult categories (fewer possible exemplars: types of music, insects, spices, criminal acts) and letters (fewer possible words beginning with the respective letter: J, K, Q, N). In a pilot study, 16 healthy young adults (who did not participate in the subsequent fMRI experiment) were asked to generate as many category exemplars or words beginning with the respective letters within one minute (i.e., a standard verbal fluency task) using the preselected stimuli. Order of presentation was randomized between subjects. As anticipated, participants produced significantly fewer exemplars or words beginning with a particular letter during the difficult conditions (easy/difficult categories: mean 21.4/12.6 correct responses; letters: 18.4/10.6 correct responses; both p<.0001).

### fMRI set-up and acquisition

Scanning was conducted at the McKnight Brain Institute (University of Florida) using a 3-Tesla Philips Achieva MR-System. For functional scanning, a T2*-weighted Fast-Field Echo, Echo-Planar-Imaging (FFE-EPI) sequence utilizing a parallel scanning technique (SENSE) was used with the following parameters: TR = 5.8 sec.; TA = 2.53 sec.; TE = 30 msec.; 38 transverse slices, interleaved acquisition, slice-thickness: 3 mm, no interslice gap; in-plane resolution: 3×3 mm; FOV: 240×240×114, acquisition matrix: 80×79. A total of 240 functional, whole brain volumes were acquired during the two sessions (80 for each of the word-generation tasks, 80 baseline volumes, total duration of the two sessions: 23.2 min). A high resolution (1×1×1 mm) anatomical scan was acquired to facilitate normalization of individual images and to ensure that participants did not have gross anatomical abnormalities. The fMRI task employed an externally paced paradigm and a temporal sparse sampling technique [11]. Overt verbal responses were assessed in the scanner during an off-phase and the hemodynamic response was acquired after a short time delay to avoid articulation related artifacts. Stimuli were presented visually by an fMRI compatible projector and a system of mirrors. Each category and letter was presented for three seconds, during which the participants responded overtly with one exemplar of the given category or a word beginning with the given letter. Afterwards, the stimulus disappeared and was replaced by a black screen (2.53 sec) and a single whole-brain functional MR volume was acquired (temporal sparse sampling). Verbal responses were transmitted from a microphone in the scanner to a speaker and transcribed. Participants were instructed to say the word ‘pass’ if they could not come up with a correct exemplar. However, other types of errors also occurred during scanning (e.g., non-responses or repetitions of the same exemplar). **Table S1** and **Table S2** provide details on numbers and types of errors produced by the participants. The distribution of errors was consistent across age-groups. Approximately 50% of errors were observed during the first two trials (i.e., at the beginning of a new task block when a new category or letter was presented) or at the end of the block (last two trials, when subjects ran out of category exemplars or words beginning with a particular letter). The remaining errors occurred during intermediate trials at variable positions.

Each condition was introduced on the visual display with a speech bubble. Afterwards, the first trial for the presented condition was displayed (i.e., semantic or phonemic word-generation, or the word ‘rest’). During scanning, alternating blocks of semantic and phonemic fluency were presented. Baseline blocks (five trials saying the word ‘rest’ aloud) were interspersed between word-generation blocks. The same categories and initial letters were used for all participants with order of appearance randomized. A training session outside of the scanner using a different set of stimuli was performed.

### Functional MRI data analysis

Pre-processing of fMRI data was performed using SPM5 (Wellcome Department of Imaging Neuroscience, London, UK). Pre-processing of data included correction for slice-time differences, spatial alignment to adjust for head movements, normalization of the functional volumes to standard MNI space and spatial smoothing with a Gaussian Kernel of 6×6×6 mm full-width-at-half-maximum. Data were modeled using a finite impulse response function [31]. The design matrix for the statistical analysis comprised the five covariates-of-interest (easy and difficult semantic or phonemic word-generation trials; baseline trials) as well as covariates-of-no-interest (movement parameters). Regressors were entered in a session specific manner and the effects of the conditions were determined in a single statistical model at the first level to account for session specific effects (e.g., different noise levels). Before estimating the modeled regressors, a high-pass filter with a cut-off period of 128 sec was applied to the data. All trials were included in the analysis. After estimation of the overall model for each participant, a random effects model on the contrast-t-maps derived from the single-subject analyses was calculated.

### Three types of analyses were conducted

(1) Regions-of-interest (ROI) in the left and right vIFG were functionally defined by a whole-brain voxelwise analysis contrasting the more difficult conditions with the easier conditions (i.e., the main effect of task difficulty). This contrast comprised data of both age-groups and both word-generation tasks. Significance level for this comparison was set to p<.005, uncorrected (voxel level) and a whole brain FWE-corrected cluster level correction of p<.05. As hypothesized, two clusters in the left and right vIFG were significantly more active during the more difficult task conditions (see **Results**). Coordinates are reported in Talairach space. (2) To further investigate how BOLD-activity levels are modulated by task difficulty across age-groups and tasks, the two vIFG clusters derived from the first analysis were used in a subsequent ROI analysis: Mean beta values from both vIFG clusters were extracted for each of the regressors (i.e., the easy and difficult semantic or phonemic word-generation conditions) for each participant. Repeated-measures ANOVAs and post-hoc paired and unpaired t-tests were used to compare activity differences in the two vIFG ROIs for each task (easy vs. difficult conditions) and within and between age-groups. (3) To assess the relationship between task-related activity in the right and left vIFG ROIs and behavioral performance, two correlation analyses were conducted: First, we assessed if absolute performance was associated with activity levels in both vIFG ROIs. Second, we correlated the individual participant's performance difference between the easy and difficult task condition (i.e., semantic task _difficult - easy_; phonemic task _difficult - easy_) with the activity differences for each task condition (i.e., semantic task _difficult – easy_; phonemic task _difficult - easy_).

## Results

### Behavioral performance (see Figure 1)

**Figure 1:**
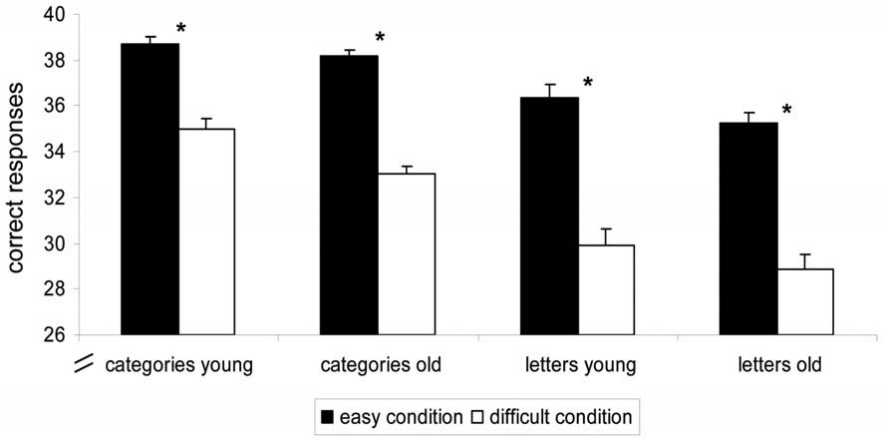
Behavioral performance. Shows absolute performance and the impact of task difficulty in the two age-groups during the semantic and phonemic task. Asterisks indicate significant differences between conditions.

Overall, participants produced fewer correct exemplars during the phonemic compared to the semantic task (main effect TASK F(1,30) = 107.47, p<.0001). Across tasks and age-groups participants produced fewer correct exemplars during the respective more difficult task conditions (mean±SD easy stimuli: 74.3±2.9; difficult stimuli: 63.4±3.6; main effect DIFFICULTY F(1,30) = 308.6, p<.0001). This difficulty effect was consistently found in both age-groups and tasks (paired t-tests: all t(15) = 7.8–16.3, all p<.0001). Overall, older subjects produced fewer correct responses across tasks and difficulty levels (main effect of AGE-GROUP: F(1,30) = 6.8, p = .02). The interaction of TASK×DIFFICULTY (F(1,30) = 14.2, p = .001) was significant. Post-hoc tests showed that the difficulty effect was more pronounced for the phonemic task in both age-groups (paired t-test across all participants: t(31) = 3.7, p = .0008).

Exploratory repeated-measures ANOVAs conducted separately for each fluency task revealed a significant AGE-GROUP×DIFFICULTY interaction only for the semantic task, indicating that the difference between the easy and difficult categories was more pronounced in the older than the younger group (F(1,30) = 4.21, p<.05; phonemic task p = .95). The older group did not perform worse on any of the categories or letters as revealed by missing interactions between the factors AGE-GROUP and CATEGORIES (F(1,7) = 0.77, p = .61) and LETTERS (F(1,7) = 0.29, p = .95; see **Table S2** for details).

### Functional activity and activity modulation

**Figure 2:**
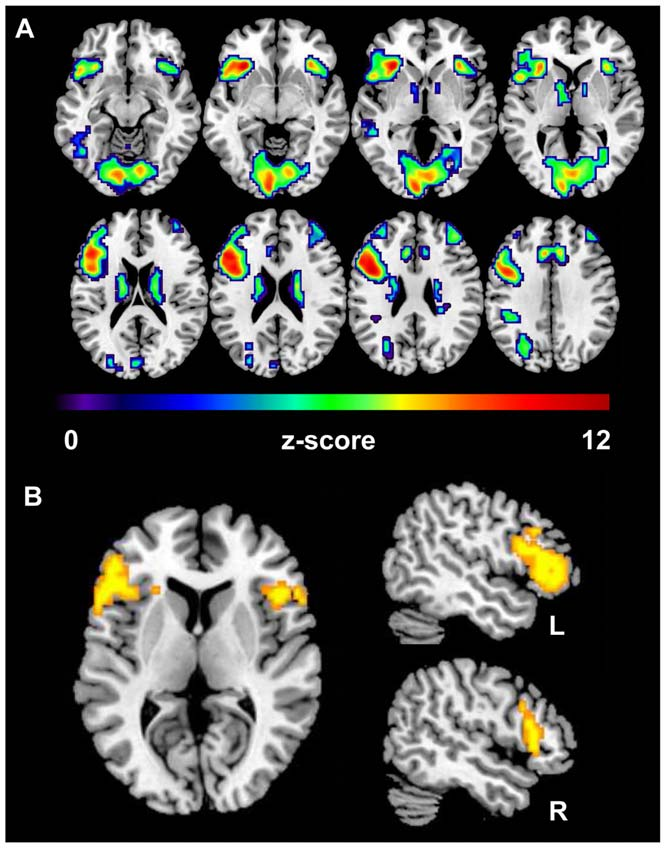
Results of the whole brain analysis. (A) Illustrates activity patterns across tasks, difficulty levels, and age-groups compared to baseline activity levels (p = .01, FWE corrected), and (B) the two clusters in the vIFG that responded to the task difficulty manipulation (p = .005, uncorrected); left = left.

**Figure 2A** illustrates the overall activity pattern elicited by the two tasks versus the baseline condition including all participants. Consistent with previous studies that used similar tasks [6], [32], a strongly left lateralized activity pattern was found with peak activity in medial and inferior frontal regions. For activity patterns associated with the two word-generation tasks and age-groups separately see **Table S3**.

The whole brain analysis that contrasted the difficult with the easy task conditions revealed that two clusters located in the left vIFG (BAs 47/45, cluster extent k = 410 voxels, Z-score 4.27, peak voxel x/y/z: −45/29/−6) and in the right vIFG (BAs 45/47, k = 120, Z-score 3.79, 48/24/10; see **Figure 2B**) were more active during the more difficult task conditions. No significant differences were found for the inverse contrast.

### ROI analysis

Repeated-measures ANOVAs, with the repeated factor TASK DIFFICULTY and the between subjects factor AGE-GROUP were conducted separately for both word-generation tasks with mean beta activity in the left and right vIFG ROIs as dependent variables. For the semantic task, a main effect of TASK DIFFICULTY was found for both vIFG ROIs (left: F(1,30) = 14.4, p = .0007; right: F(1,30) = 10.2, p = .003), indicating that in both age-groups and vIFG ROIs mean beta values were higher during the more difficult task conditions. Except for the right vIFG in the younger group, these activity increases were significant (post-hoc paired t-tests: left vIFG: t(15) = 2.37, p = .01; right vIFG t(15) = .94, p = .18; old: left vIFG: t(15) = 2.97, p = .004; right vIFG: t(15) = 3.28, p = .002). No interaction between TASK DIFFICULTY and AGE-GROUP (p = .58) was found for the left vIFG ROI and there was no main effect for the factor AGE-GROUP (p = .38). However, for the right vIFG ROI a significant interaction of TASK DIFFICULTY and AGE-GROUP was found (F(1,30) = 4.2, p = 0.04 and there was a trend for more pronounced activity in the right vIFG ROI in the older group (main effect GROUP: right vIFG (F(1,30) = 3.1, p = .08). This was explained by more pronounced activity in the older group during the semantic task (difficult condition t(30) = 2.53, p = .02; easy condition t(30) = .19, p = .84). Thus, during the task condition that was disproportionately difficult for the old compared to the young group, these subjects also showed more pronounced modulation of right vIFG activity relative to the young group.

For the phonemic task, a main effects of TASK DIFFICULTY were found in both vIFG ROIs (left: F(1,30) = 13.3, p = .001; right: F(1,30) = 13.0, p = .001), however, no significant interactions between TASK DIFFICULTY and AGE-GROUP emerged (both vIFG ROIs p>.6). Activity was comparable between the two age-groups in the left vIFG ROI for both difficulty levels (main effect AGE-GROUP: p = .43), however, older subjects had more pronounced activity in the right vIFG ROI as indicated by a significant main effect of AGE-GROUP (F(1,30) = 5.57, p = .02).

In addition, we compared mean beta activity elicited by the (easier) semantic and (more difficult) phonemic tasks in both vIFG ROIs. Across age-groups and difficulty levels, no differences between the two tasks were found in the left ROI (t(63), p = .80); however, in the right vIFG ROI, activity levels were higher for the more demanding phonemic task (phonemic>semantic task (t(63) = 2.78, p = .007).

### Correlation of bilateral vIFG activity and performance

**Figure 3:**
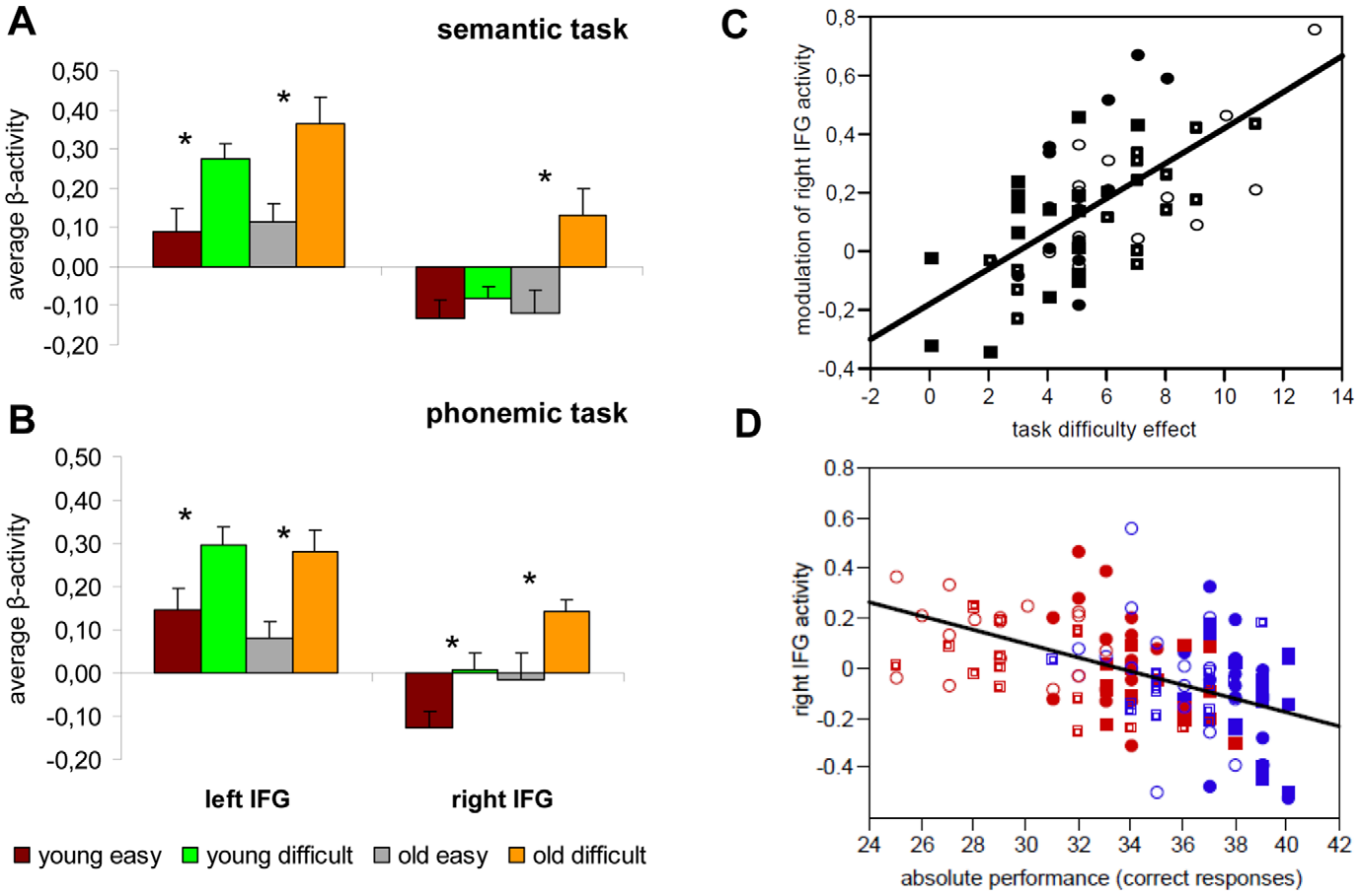
Region of interest analysis. Illustrates the modulation of activity bilaterally in vIFG clusters by task difficulty during the (A) semantic and (B) phonemic task for age-group (young vs. old) and hemisphere (left vs. right). Stars indicate difficulty comparisons that are significant at the various levels of TASK, AGE-GROUP, and HEMISPHERE. (C) Positive correlation of individual task difficulty (difficult - easy) with modulation of individual activity levels in the right vIFG (difficult - easy). (D) Negative correlation of right vIFG activity with absolute performance accuracy across age-groups, fluency tasks, and difficulty conditions [easy conditions = blue; difficult conditions = red; circles = older group; squares = younger group; solid = semantic fluency, open = phonemic fluency].

Overall, task difficulty, expressed as individual performance difference between difficult and easy conditions across both age-groups and tasks was correlated with increased activity for difficult compared to easy items in both vIFG clusters (left vIFG r = .46, p = .0001; right vIFG r = .60, p<.0001). Thus, irrespective of age, more pronounced differences between the easy and difficult task conditions were associated with increased activity in these areas (**Figure 3C**). This overall linear trend could be confirmed for both vIFG clusters, age-groups, and fluency tasks separately (all r = .55−.80, p = .02−.0002), except for the left vIFG in the young group during the semantic task (r = .46, p = .07).

Moreover, activity in both vIFG clusters was negatively correlated with performance across age-groups, fluency tasks, and conditions (left vIFG: r = −.42, p<.0001; right vIFG: r = −.47, p<.0001; **see** **Figure 3D**), i.e., more pronounced activity in left and right vIFG predicted fewer correct responses across tasks and age-groups. Again, this could be confirmed for both vIFG ROIs, age-groups, and tasks (all r = −.42–.59, p = .016−.0003), except for the right vIFG in the young group during the semantic task (r = −.29, p = .096).

The correlation strength between (a) performance and absolute activity and (b) performance differences and activity modulation was comparable in the two age-groups (Fisher r-to-z transformation [33]: absolute performance vs. absolute activity: left IFG z = .04, p = .96, right IFG z = −.01, p = .99; performance difference vs. activity modulation: left IFG z = −.05, p = .96, right IFG z = .47, p = .64).

## Discussion

The study was motivated by the fact that previous functional imaging studies using word-retrieval tasks found enhanced activity in prefrontal areas of the non-dominant hemisphere in older adults, but did not systematically control for performance-related factors. Thus, in the present study we manipulated difficulty levels during semantic and phonemic word-generation and assessed functional activity in the ventral portion of the IFG, an area associated with controlled selection processes, and reliably activated during semantic and phonemic tasks [10], [15].

We show that despite regional differences in BOLD activity between older and younger adults at a given difficulty level, these differences are crucially mediated by performance accuracy. In particular, across age-groups and word-generation tasks, two regions in the left and right vIFG showed an increase of activity during the more difficult task conditions. In addition, we found linear correlations between absolute activity in the vIFG bilaterally and performance accuracy, i.e., more pronounced activity was associated with reduced performance across age-groups, tasks, and difficulty levels. Moreover, performance differences between the easy and difficult task conditions were linearly correlated with activity increases in both ROIs, across tasks and age-groups. Thus, in line with previous reports in other cognitive domains [9], differences in functional activation are not solely explained by chronological age, but crucially mediated by performance accuracy. Enhanced bilateral vIFG activity during the more difficult task conditions cannot be interpreted as “compensatory”, as it was associated with reduced performance. Moreover, it cannot solely be explained by structural deterioration of grey or white matter structures in the older group, as bilateral vIFG activity increases were found in both age-groups. Thus, increased bilateral activity is best explained by enhanced demands placed on top-down control processes (i.e., semantic search and selection) during the more difficult task conditions. Moreover, control processes may have been more challenged in the older group due to deterioration of specialized neural populations in left frontal areas [4] or medial temporal structures [34], possibly explaining why older adults show more pronounced activity in the right vIFG even at lower levels of task difficulty. We will discuss our findings and their implications in more detail below.

In line with a number of previous studies [6], [16], [35], younger and older adults produced fewer correct words during phonemic compared to semantic word-generation, possibly related to a more “natural” search strategy based on semantic relations during the semantic task [36]. Furthermore, subjects of both age-groups consistently produced fewer correct responses during the more difficult conditions in both the phonemic and semantic tasks. Overall, this difficulty effect was more pronounced in the older group, despite careful matching of participants for demographic variables. Similar results have been reported in a number of previous studies [35], [37]. Extensive neuropsychological testing assured high levels of cognitive functioning in our group of older participants. However, reduced performance during tasks targeting executive functions like word-generation, has frequently been reported in healthy aging [4]. In addition, most studies that compared groups of younger and healthy older adults found selectively impaired semantic but not phonemic word-generation in the older groups [35], [37]. Interestingly, similar findings have been reported in patients with Alzheimer's disease or Mild Cognitive Impairment [1], [16] and explained by impaired functioning of the medial temporal lobe in these patients. Thus, changes in hippocampal functions [34] may also explain selectively impaired semantic word-generation in healthy older adults. In our own study this was expressed as a relatively more pronounced drop in performance during the semantic compared to the phonemic task and more pronounced activity increases in the right vIFG.

As hypothesized, two clusters in the left and right ventral portion of the IFG showed increased BOLD activity during the difficult as compared to the easy task conditions. Absolute activity and activity modulation by task difficulty in the left vIFG cluster was comparable between age-groups for both word-generation tasks, and superior performance was associated with less pronounced positive task-related activity. A different picture emerged for the right vIFG. In line with previous studies in healthy younger and older adults [8], [38], the right vIFG cluster was not active during the less demanding semantic task, except for the more difficult semantic task in the older group. Instead, strong negative task-related activity was found. More pronounced activity increases in the older group during the more difficult semantic condition were explained by relatively greater drop in performance during this condition than for the younger group. During the generally more demanding phonemic task, right vIFG activity was less negative or even positive, except for the easy phonemic task in the young group. However, the relative modulation by task difficulty was again comparable between age-groups. Thus, the right vIFG was “deactivated” in younger participants, except for the most demanding task condition (i.e., the more difficult phonemic task). In contrast, it was already up-regulated in older adults during the more difficult semantic task which was associated with significantly decreased performance accuracy. Thus, our findings are in line with a number of previous studies in other cognitive domains showing that prefrontal areas in the non-task dominant hemisphere (that are up-regulated in younger adults only during more demanding task conditions) are more active in older adults at lower levels of difficulty [4].

From a methodological point of view, the design allowed us to compare performance-associated within-group activity modulations which were similar in both age-groups. This finding makes it unlikely that differences of absolute activity between age-groups are simply explained by changes in hemodynamic properties in the older groups [9]. Moreover, in line with a recent quantitative meta-analysis [39] and results in the working memory domain [9], older participants exhibiting a ‘more youth-like pattern’ (i.e., less pronounced activity in both prefrontal ROIs) performed better during word-generation, and younger adults who performed poorer exhibited more pronounced activity in the left and right vIFG. Our findings are also in line with functional imaging studies of language development [40] and second-language acquisition [41] and very recent studies that applied non-invasive brain stimulation to the left IFG during language production tasks [32], [42], showing that efficient and focal processing in prefrontal cortices is associated with better performance.

In the present study, we used a paced block design and a sparse temporal sampling procedure with a short inter-stimulus interval (TR∼5.5 sec) and included all trials in the analysis in order to allow for a comparison of the present study with previous studies that used a similar methodology [6], [11]. A possible alternative would have been an event-related design with a very long TR, where the hemodynamic response can return to baseline after each trial, and erroneous trials can be analyzed separately. We opted against such a design as this would have significantly increased the duration of the experiment and also resulted in a very non-natural type of verbal fluency task due to the long inter-stimulus interval. However, it needs to be acknowledged that the paced design of the present study may have resulted in enhanced processing demands compared to conventional verbal fluency paradigms. Indeed, previous studies that compared paced and unpaced paradigms found more pronounced activity in areas associated with sustained attention, motor planning and response inhibition [43]. This may also explain more pronounced differences between younger and older adults during the intrascanner semantic task compared to the out-of-scanner task. In addition, due to our experimental design (block design) and the relatively small (and variable) number of erroneous trials, we are confident that the reported activity differences between the respective task conditions are related to increased task demands during word-retrieval. This notion receives support from a recent study in which an event-related design and a picture naming task (i.e., a different type of word-retrieval task) were used to differentiate activity related to correct and erroneous word-retrieval [44]. The authors report activity patterns for correct and erroneous trials to be “strikingly similar” (p. 167) and the number of errors correlated with increased activity in the middle and medial frontal gyrus, but not the vIFG. Moreover, we recently used a similar fMRI design to assess the impact of excitatory (anodal) transcranial direct current stimulation (atDCS) on semantic word-generation in 20 healthy younger adults [32]. In line with the results of the present study, we found that improved semantic word-generation during atDCS compared to a placebo (“sham”) stimulation condition was associated with selectively reduced activity in the ventral portion of the IFG.

Our present study was not specifically designed to differentiate between the many cognitive processes that may have been affected by the task difficulty manipulation. For example, while both tasks require a strategic search and controlled retrieval of information, phonemic word-generation may be facilitated by automatic activation of semantic operations and semantic tasks require engagement of low-level phonological processes. Furthermore, both tasks involve additional cognitive operations that are neither phonological nor semantic per se (e.g., working memory-related) [12], [14]. On the other hand, there are differences between the two word-generation tasks with regard to cognitive operations and neural systems supporting these functions. For example, semantic generation relies on semantic associations within a category, whereas phonemic fluency may be accomplished with a relatively less constrained search from a broader set of lexical exemplars [14]. Thus, our data do not allow for definitive conclusions about specific processes that may explain increased activity in the left and right IFG. Rather, our main analysis was designed to elucidate which areas respond in a similar way to a manipulation of task difficulty across age-groups and word-generation tasks. Our findings may also not generalize to other tasks or brain regions, although previous studies that used non-language tasks (e.g., working memory tasks) found differences in activity and activity modulation between age-groups in more anterior dorsolateral prefrontal cortices [3], [39].

In sum, the results of the present study show that age-related differences in functional activity during word-retrieval in the vIFG are crucially mediated by performance accuracy. While this confirms previous findings showing that activity differences during word-retrieval in the prefrontal cortex may be associated with impaired performance rather than aging per se [6], [11], this does not preclude the possibility that other brain regions may be modulated differentially in younger and older adults [7]. Our findings need to be taken into account in future functional imaging studies in healthy aging and may also be of relevance when imaging pathological aging processes like post-stroke language impairments (aphasia) [45], [46] or prodromal stages of Alzheimer's disease [47]. In these conditions, increased brain activity in prefrontal areas has mainly been interpreted as a consequence of neural reorganization in response to brain pathology, however, differences between patients and controls may also be mediated by task-difficulty effects.

## Supporting Information

Table S1 Average number of correct responses and errors in both age-groups (mean and standard deviation, max. 40 correct responses or errors, respectively).
(DOC)

Table S2 Average number of correct responses for categories and letters for both age-groups (max. 10 correct responses).
(DOC)

Table S3 Shows details of the activity patterns associated with the two word-generation tasks for the both age-groups and task-difficulty conditions [peak voxels in significant clusters (p<.05, FEW-corrected) are reported; cluster extent k>10; voxel threshold: p<.005 FDR-corrected].
(DOC)
