# Supplementary material for: Same Modulation but Different Starting Points: Performance Modulates Age Differences in Inferior Frontal Cortex Activity during Word-Retrieval
Source: PLoS One. 2012 Mar 15;7(3):e33631. doi: 10.1371/journal.pone.0033631 (PMC3305312; doi:10.1371/journal.pone.0033631)
Supplement: Table S1 — Average number of correct responses and errors in both age-groups (mean and standard deviation, max. 40 correct responses or errors, respectively). (DOC) [file pone.0033631.s001.doc]

**Table S1**

|  | **easy categories** | | | | | **difficult categories** | | | | | **easy letters** | | | | | **difficult letters** | | | | |
| --- | --- | --- | --- | --- | --- | --- | --- | --- | --- | --- | --- | --- | --- | --- | --- | --- | --- | --- | --- | --- |
|  |  | error types | | | |  | error types | | | |  | error types | | | |  | error types | | | |
| **YOUNG GROUP** | **correct responses** | 1 | 2 | 3 | 4 | **correct responses** | 1 | 2 | 3 | 4 | **correct responses** | 1 | 2 | 3 | 4 | **correct responses** | 1 | 2 | 3 | 4 |
| mean | **38.69** | **1.00** | **0.25** | **0.06** | **0.00** | **35.00** | **2.94** | **1.56** | **0.25** | **0.31** | **36.38** | **1.88** | **1.19** | **0.13** | **0.38** | **29.94** | **6.00** | **2.94** | **0.69** | **0.44** |
| SD | 1.25 | 1.15 | 0.58 | 0.25 | 0.00 | 1.71 | 1.48 | 1.21 | 0.58 | 0.70 | 2.39 | 1.36 | 1.52 | 0.34 | 0.72 | 2.82 | 2.03 | 1.77 | 1.14 | 0.89 |
|  |  |  |  |  |  |  |  |  |  |  |  |  |  |  |  |  |  |  |  |  |
| **OLD GROUP** | **correct responses** | 1 | 2 | 3 | 4 | **correct responses** | 1 | 2 | 3 | 4 | **correct responses** | 1 | 2 | 3 | 4 | **correct responses** | 1 | 2 | 3 | 4 |
| mean | **38.19** | **1.06** | **0.63** | **0.06** | **0.06** | **33.06** | **5.00** | **1.63** | **0.19** | **0.13** | **35.25** | **2.94** | **1.63** | **0.13** | **0.06** | **28.88** | **6.38** | **3.81** | **0.50** | **0.44** |
| SD | 1.05 | 0.93 | 0.62 | 0.25 | 0.25 | 1.18 | 1.21 | 0.89 | 0.54 | 0.34 | 1.84 | 1.29 | 1.26 | 0.34 | 0.25 | 2.60 | 1.54 | 1.47 | 0.63 | 1.09 |

error types: 1=subjects said "pass". 2=omissions (non-responses). 3=repetitions (including synonyms). 4=errors (e.g.. words that do not belong to the given category or do not begin with the given letter)
