# Supplementary material for: Same Modulation but Different Starting Points: Performance Modulates Age Differences in Inferior Frontal Cortex Activity during Word-Retrieval
Source: PLoS One. 2012 Mar 15;7(3):e33631. doi: 10.1371/journal.pone.0033631 (PMC3305312; doi:10.1371/journal.pone.0033631)
Supplement: Table S2 — Average number of correct responses for categories and letters for both age-groups (max. 10 correct responses). (DOC) [file pone.0033631.s002.doc]

**Table S2**

|  | **CATEGORIES** | | | | | | | |  | **LETTERS** | | | | | | | |
| --- | --- | --- | --- | --- | --- | --- | --- | --- | --- | --- | --- | --- | --- | --- | --- | --- | --- |
|  | **body parts** | **colors** | **clothing** | **beverages** | **spices** | **criminal acts** | **insects** | **musical instruments** |  | **M** | **S** | **T** | **R** | **J** | **Q** | **N** | **K** |
|  |  | | | | | | | | | | | | | | | | |
|  | **YOUNG GROUP** | | | | | | | | | | | | | | | | |
| **mean** | **9.75** | **9.56** | **9.75** | **9.63** | **8.56** | **8.56** | **8.94** | **8.94** |  | **8.56** | **9.44** | **9.13** | **9.25** | **8.06** | **7.00** | **8.38** | **6.50** |
| **SD** | 0.45 | 0.51 | 0.45 | 0.50 | 0.96 | 0.81 | 0.68 | 0.93 |  | 1.21 | 0.63 | 0.72 | 0.93 | 1.06 | 1.63 | 1.31 | 1.46 |
|  |  | | | | | | | | | | | | | | | | |
|  | **OLD GROUP** | | | | | | | | | | | | | | | | |
| **mean** | **9.81** | **9.44** | **9.56** | **9.38** | **8.06** | **8.13** | **8.56** | **8.31** |  | **8.38** | **9.31** | **8.94** | **8.63** | **7.56** | **6.75** | **8.06** | **6.50** |
| **SD** | 0.40 | 0.51 | 0.51 | 0.62 | 1.18 | 0.62 | 1.03 | 0.70 |  | 0.81 | 0.70 | 0.68 | 0.62 | 1.09 | 1.29 | 1.06 | 0.89 |
