# Supplementary material for: Same Modulation but Different Starting Points: Performance Modulates Age Differences in Inferior Frontal Cortex Activity during Word-Retrieval
Source: PLoS One. 2012 Mar 15;7(3):e33631. doi: 10.1371/journal.pone.0033631 (PMC3305312; doi:10.1371/journal.pone.0033631)
Supplement: Table S3 — Shows details of the activity patterns associated with the two word-generation tasks for the both age-groups and task-difficulty conditions [peak voxels in significant clusters (p<.05, FEW-corrected) are reported; cluster extent k>10; voxel threshold: p<.005 FDR-corrected]. (DOC) [file pone.0033631.s003.doc]

**Table S3**

| **YOUNG GROUP** | | | | | | | | | | | | | | | | |
| --- | --- | --- | --- | --- | --- | --- | --- | --- | --- | --- | --- | --- | --- | --- | --- | --- |
| **semantic task > baseline** | | | | | | | | | | | | | | | | |
| **easy condition** | | | | | | | |  | **difficult condition** | | | | | | | |
| **Anatomical structure** | **Hemi** | **BA** | **k** | **Z** | **x** | **y** | **z** |  | **Anatomical structure** | **Hemi** | **BA** | **k** | **Z** | **x** | **y** | **z** |
| **Superior frontal gyrus** | L | 6 | 551 | 7.82 | -3 | 14 | 55 |  | **Superior Frontal Gyrus** | L | 6 | 483 | 7.30 | 0 | 14 | 45 |
| Cingulate gyrus | L | 32 |  | 7.21 | -6 | 19 | 38 |  | Medial Frontal Gyrus | L | 8 |  | 6.70 | 0 | 23 | 43 |
| Medial frontal gyrus | L | 6 |  | 7.17 | -6 | 14 | 44 |  | Cingulate Gyrus | L | 32 |  | 6.60 | -6 | 19 | 38 |
| **Middle Frontal Gyrus** | L | 9 | 711 | 6.90 | -45 | 13 | 24 |  | **Lingual Gyrus** | L | 18 | 872 | 6.60 | -6 | -85 | -1 |
| Inferior Frontal Gyrus | L | 47 |  | 6.52 | -30 | 23 | -6 |  |  | R | 18 |  | 5.55 | 12 | -82 | -9 |
| Insula | L | 13 |  | 6.18 | -33 | 18 | 2 |  |  |  |  |  |  |  |  |  |
| **Lingual Gyrus** | L | 18 | 415 | 6.00 | -12 | -79 | -4 |  | **Inferior Frontal Gyrus** | L | 9 | 723 | 6.59 | -50 | 16 | 24 |
| Cuneus | L | 17 |  | 5.86 | -9 | -90 | 2 |  |  | L | 45 |  | 6.33 | -50 | 24 | 13 |
| Lingual Gyrus | R | 18 |  | 5.12 | 12 | -82 | -9 |  | Middle Frontal Gyrus | L | 46 |  | 6.32 | -45 | 21 | 21 |
| **Inferior Temporal Gyrus** | L | 20 | 57 | 5.32 | -50 | -53 | -12 |  | **Caudate Nucleus** | R |  | 62 | 5.02 | 15 | -16 | 20 |
| Fusiform Gyrus | L | 37 |  | 4.13 | -48 | -62 | -15 |  |  |  |  |  |  |  |  |  |
| **Insula** | R | 13 | 37 | 5.13 | 33 | 23 | -1 |  | **Middle Frontal Gyrus** | L | 6 | 48 | 4.88 | -24 | -1 | 44 |
| **Superior Parietal Lobule** | L | 7 | 80 | 5.03 | -27 | -68 | 42 |  | **Precuneus** | L | 19 | 83 | 4.72 | -30 | -65 | 42 |
|  |  |  |  |  |  |  |  |  | Superior Parietal Lobule | L | 7 |  | 3.94 | -21 | -64 | 56 |
| **Caudate Nucleus** | R |  | 33 | 4.57 | 18 | -5 | 20 |  | **Caudate** | L |  | 48 | 4.64 | -15 | -2 | 17 |
| **Thalamus** | L |  | 54 | 4.37 | -15 | -11 | 17 |  | **Precuneus** | L | 7 | 21 | 4.59 | -21 | -50 | 41 |
| Putamen | L |  |  | 4.31 | -15 | 6 | 8 |  |  |  |  |  |  |  |  |  |
| **Middle Frontal Gyrus** | L | 46 | 22 | 4.35 | -39 | 47 | 17 |  | **Inferior Temporal Gyrus** | L | 37 | 60 | 4.36 | -50 | -39 | -18 |
| Superior Frontal Gyrus | L | 10 |  | 3.92 | -30 | 45 | 17 |  |  | L | 20 |  | 4.12 | -50 | -50 | -13 |
| **Superior Frontal Gyrus** | L | 8 | 19 | 4.34 | -30 | 20 | 49 |  | **Thalamus** | L |  | 28 | 4.15 | -3 | -20 | 12 |
|  |  |  |  |  |  |  |  |  | **Parahippocampal Gyrus** | R | 30 | 24 | 4.12 | 27 | -55 | 6 |
|  | | | | | | | | | | | | | | | | |
| **phonemic task > baseline** | | | | | | | | | | | | | | | | |
| **easy condition** | | | | | | | |  | **difficult condition** | | | | | | | |
| **Anatomical structure** | **Hemi** | **BA** | **k** | **Z** | **x** | **y** | **z** |  | **Anatomical structure** | **Hemi** | **BA** | **k** | **Z** | **x** | **y** | **z** |
| **Superior Frontal Gyrus** | L | 6 | 615 | >9 | -3 | 14 | 55 |  | **Middle Frontal Gyrus** | L | 9 | 1832 | 7.78 | -45 | 13 | 24 |
| Medial Frontal Gyrus | L | 32 |  | 7.40 | -6 | 11 | 46 |  | Superior Frontal Gyrus | L | 6 |  | 7.49 | -3 | 14 | 55 |
| Cingulate Gyrus | L | 32 |  | 7.19 | -3 | 22 | 38 |  | Cingulate Gyrus | L | 32 |  | 7.44 | -6 | 22 | 38 |
| **Inferior Frontal Gyrus** | L | 9 | 994 | 7.10 | -48 | 13 | 24 |  | **Inferior Frontal Gyrus** | R | 47 | 106 | 5.99 | 33 | 26 | -6 |
|  | L | 47 |  | 6.73 | -30 | 23 | -6 |  | Inferior Frontal Gyrus | R | 13 |  | 3.83 | 45 | 27 | 10 |
|  | L | 44 |  | 6.63 | -50 | 12 | 16 |  |  |  |  |  |  |  |  |  |
| **Lingual Gyrus** | L | 17 | 387 | 6.15 | -9 | -93 | 0 |  | **Lingual Gyrus** | L | 17 | 154 | 5.10 | -12 | -93 | 2 |
| Cuneus | L | 23 |  | 4.74 | -6 | -75 | 9 |  |  | L | 18 |  | 4.10 | -9 | -79 | -6 |
| Lingual Gyrus | L | 18 |  | 4.73 | -15 | -67 | -4 |  | Middle Occipital Gyrus | L | 18 |  | 4.10 | -21 | -82 | -6 |
| **Inferior Frontal Gyrus** | R | 47 | 112 | 5.45 | 33 | 26 | -1 |  | **Inferior Parietal Lobule** | L | 40 | 40 | 4.82 | -42 | -38 | 46 |
| **Fusiform Gyrus** | L | 37 | 62 | 5.18 | -48 | -59 | -12 |  | **Superior Parietal Lobule** | L | 7 | 54 | 4.74 | -27 | -68 | 48 |
|  |  |  |  |  |  |  |  |  | Precuneus | L | 7 |  | 4.22 | -24 | -68 | 34 |
| **Inferior Parietal Lobule** | L | 40 | 50 | 5.12 | -39 | -36 | 38 |  | **Putamen** | L |  | 46 | 4.57 | -15 | 3 | 11 |
|  |  |  |  |  |  |  |  |  | Caudate Nucleus | L |  |  | 4.13 | -18 | 10 | 16 |
| **Parahippocampal Gyrus** | R | 30 | 36 | 4.98 | 30 | -55 | 6 |  | **Parahippocampal Gyrus** | R | 30 | 26 | 4.53 | 30 | -55 | 6 |
|  |  |  |  |  |  |  |  |  | Lingual Gyrus | R | 19 |  | 3.79 | 30 | -64 | 6 |
| **Middle Temporal Gyrus** | L | 22 | 59 | 4.73 | -53 | -35 | 2 |  | **Posterior Cingulate** | L | 30 | 20 | 4.41 | -27 | -67 | 9 |
| **Thalamus** | R |  | 172 | 4.58 | 3 | -23 | 15 |  | **Fusiform Gyrus** | L | 37 | 31 | 4.40 | -48 | -59 | -12 |
| Putamen | L |  |  | 4.57 | -18 | 6 | 11 |  |  |  |  |  |  |  |  |  |
| Caudate Nucleus | L |  |  | 4.36 | -12 | -8 | 20 |  |  |  |  |  |  |  |  |  |
| **Caudate** | R |  | 71 | 4.57 | 21 | -5 | 22 |  | **Middle Temporal Gyrus** | L | 22 | 20 | 4.17 | -48 | -38 | 2 |
| **Lingual Gyrus** | R | 18 | 20 | 4.43 | 21 | -79 | -4 |  |  |  |  |  |  |  |  |  |
| **Middle Frontal Gyrus** | L | 6 | 26 | 4.37 | -24 | -1 | 47 |  |  |  |  |  |  |  |  |  |

R = right, L = left; Hemi = hemisphere; BA = Brodman area; k = cluster extent (voxels); Z = Z-value; x/y/z = coordinates of peak voxels in significant clusters in Talairach space; bold = peak voxel in significant cluster

| **OLD GROUP** | | | | | | | | | | | | | | | | |
| --- | --- | --- | --- | --- | --- | --- | --- | --- | --- | --- | --- | --- | --- | --- | --- | --- |
| **semantic task > baseline** | | | | | | | | | | | | | | | | |
| **easy condition** | | | | | | | |  | **difficult condition** | | | | | | | |
| **Anatomical structure** | **Hemi** | **BA** | **k** | **Z** | **x** | **y** | **z** |  | **Anatomical structure** | **Hemi** | **BA** | **k** | **Z** | **x** | **y** | **z** |
| **Inferior Frontal Gyrus** | L | 9 | 7192 | 7.76 | -42 | 7 | 27 |  | **Cingulate Gyrus** | L | 32 | 4166 | 7.05 | -6 | 19 | 38 |
| Lingual Gyrus | R | 18 |  | 7.23 | 12 | -79 | -4 |  | Inferior Frontal Gyrus | L | 9 |  | 6.89 | -39 | 7 | 27 |
| Cingulate Gyrus | L | 32 |  | 7.15 | -6 | 19 | 38 |  | Medial Frontal Gyrus | L | 32 |  | 6.44 | -9 | 14 | 46 |
| **Middle Frontal Gyrus** | R | 46 | 162 | 5.56 | 42 | 42 | 26 |  | **Lingual Gyrus** | R | 18 | 1597 | 6.50 | 15 | -76 | -6 |
| Superior Frontal Gyrus | R | 10 |  | 4.25 | 36 | 53 | 14 |  |  | L | 18 |  | 5.99 | -9 | -82 | -1 |
| **Inferior Frontal Gyrus** | R | 47 | 101 | 5.01 | 33 | 26 | -6 |  | **Middle Frontal Gyrus** | R | 46 | 170 | 4.89 | 42 | 42 | 26 |
|  |  |  |  | 4.08 | 48 | 17 | -6 |  |  |  |  |  |  |  |  |  |
| **Anterior Cingulate Gyrus** | R |  | 45 | 4.30 | 6 | 27 | 11 |  | **Precuneus** | L | 7 | 171 | 4.88 | -18 | -53 | 55 |
|  |  |  |  |  |  |  |  |  | Superior Parietal Lobule | L | 7 |  | 4.14 | -27 | -68 | 48 |
|  |  |  |  |  |  |  |  |  | **Inferior Parietal Lobule** | L | 40 | 61 | 4.35 | -48 | -33 | 38 |
|  | | | | | | | | | | | | | | | | |
| **phonemic task > baseline** | | | | | | | | | | | | | | | | |
| **easy condition** | | | | | | | |  | **difficult condition** | | | | | | | |
| **Anatomical structure** | **Hemi** | **BA** | **k** | **Z** | **x** | **y** | **z** |  | **Anatomical structure** | **Hemi** | **BA** | **k** | **Z** | **x** | **y** | **z** |
| **Inferior Frontal Gyrus** | L | 9 | 1279 | 6.83 | -42 | 7 | 27 |  | **Inferior Frontal Gyrus** | L | 9 | 2902 | 7.60 | -42 | 7 | 25 |
| Superior Frontal Gyrus | L | 6 |  | 6.09 | -6 | 11 | 49 |  | Superior Frontal Gyrus | R | 6 |  | 6.72 | 7 | 11 | 49 |
| Medial Frontal Gyrus | R | 6 |  | 5.95 | 6 | 14 | 44 |  | Precentral Gyrus | L | 6 |  | 6.47 | -48 | 2 | 47 |
| **Lingual Gyrus** | R | 18 | 1641 | 5.58 | 9 | -73 | -6 |  | **Middle Occipital Gyrus** | L | 19 | 2260 | 6.21 | -53 | -61 | -4 |
|  | R | 19 |  | 5.53 | 33 | -64 | 3 |  | Inferior Parietal Lobule | L | 40 |  | 6.21 | -48 | -30 | 32 |
|  | L | 17 |  | 5.47 | -6 | -90 | -1 |  | Cuneus | L | 17 |  | 5.40 | -9 | -90 | 2 |
| **Thalamus** | L |  | 146 | 5.23 | -15 | -11 | 12 |  | **Middle Frontal Gyrus** | R | 10 | 125 | 5.80 | 39 | 45 | 25 |
|  |  |  |  |  |  |  |  |  | Superior Frontal Gyrus | R | 10 |  | 5.43 | 36 | 56 | 14 |
| **Insula** | R | 13 | 64 | 5.18 | 42 | 7 | 13 |  | **Inferior Frontal Gyrus** | R | 47 | 259 | 5.51 | 45 | 17 | -3 |
| Middle Frontal Gyrus | R | 9 |  | 3.76 | 48 | 13 | 30 |  | Insula | R | 13 |  | 4.82 | 33 | 23 | -1 |
| Inferior Frontal Gyrus | R | 44 |  | 3.46 | 50 | 15 | 16 |  | Inferior Frontal Gyrus | R | 45 |  | 4.71 | 48 | 18 | 16 |
| **Cingulate Gyrus** | R | 31 | 283 | 5.08 | 18 | -34 | 27 |  | **Parahippocampal Gyrus** | L |  | 52 | 4.53 | -27 | -18 | -9 |
|  |  |  |  |  |  |  |  |  | Putamen | L |  |  | 3.86 | -27 | -20 | -1 |
| **Inferior Parietal Lobule** | L | 40 | 64 | 4.88 | -48 | -33 | 38 |  |  |  |  |  |  |  |  |  |
| **Posterior Cingulate Gyrus** | L | 29 | 59 | 4.75 | -15 | -40 | 13 |  |  |  |  |  |  |  |  |  |
| **Inferior Frontal Gyrus** | R | 47 | 99 | 4.56 | 33 | 26 | -4 |  |  |  |  |  |  |  |  |  |
| **Middle Frontal Gyrus** | R | 6 | 105 | 4.40 | 30 | 2 | 44 |  |  |  |  |  |  |  |  |  |
| Superior Frontal Gyrus | R | 6 |  | 4.22 | 21 | 6 | 61 |  |  |  |  |  |  |  |  |  |
| **Middle Frontal Gyrus** | R | 10 | 35 | 4.31 | 42 | 45 | 23 |  |  |  |  |  |  |  |  |  |
| **Caudate Nucleus** | R |  | 31 | 4.30 | 21 | -2 | 25 |  |  |  |  |  |  |  |  |  |
| Thalamus | R |  |  | 4.15 | 15 | -8 | 11 |  |  |  |  |  |  |  |  |  |
| **Posterior Cingulate Gyrus** | R | 29 | 26 | 4.22 | 15 | -40 | 16 |  |  |  |  |  |  |  |  |  |

R = right, L = left; Hemi = hemisphere; BA = Brodman area; k = cluster extent (voxels); Z = Z-value; x/y/z = coordinates of peak voxels in significant clusters in Talairach space; bold = peak voxel in significant cluster
